# Formation of hydrated PEG layers on magnetic iron oxide nanoflowers shows internal magnetisation dynamics and generates high in-vivo efficacy for MRI and magnetic hyperthermia

**DOI:** 10.1016/j.actbio.2022.08.033

**Published:** 2022-08-23

**Authors:** Eoin P. McKiernan, Cara Moloney, Tista Roy Chaudhuri, Shane Clerkin, Kevin Behan, Robert M. Straubinger, John Crean, Dermot F. Brougham

**Affiliations:** a School of Chemistry, University College Dublin, Belfield, Dublin 4, Ireland; b Department of Pharmaceutical Sciences, School of Pharmacy and Pharmaceutical Sciences, University at Buffalo, State University of New York, Buffalo, NY, 14214, USA; c School of Biomolecular and Biomedical Science, Conway Institute, University College Dublin, Belfield, Dublin 4, Ireland; d Department of Cell Stress Biology Roswell Park Comprehensive Cancer Center, Buffalo, NY 14263, USA; e School of Medicine, University of Nottingham, NG7 2RD, UK

**Keywords:** Iron oxide, Multicore nanoparticles, Polyethylene glycol, Relaxivity, MRI contrast agent, Magnetic hyperthermia

## Abstract

Multicore magnetic iron oxide nanoparticles, nanoflowers (NFs), have potential biomedical applications as efficient mediators for AC-magnetic field hyperthermia and as contrast agents for magnetic resonance imaging due to their strong magnetic responses arising from complex internal magnetic ordering. To realise these applications amenable surface chemistry must be engineered that maintain particle dispersion. Here a catechol-derived grafting approach is described to strongly bind polyethylene glycol (PEG) to NFs and provide stable hydrogen-bonded hydrated layers that ensure good long-term colloidal stability in buffers and media even at clinical MRI field strength and high concentration. The approach enables the first comprehensive study into the MRI (relaxivity) and hyperthermic (SAR) efficiencies of fully dispersed NFs. The predominant role of internal magnetisation dynamics in providing high relaxivity and SAR is confirmed, and it is shown that these properties are unaffected by PEG molecular weight or corona formation in biological environments. This result is in contrast to traditional single core nanoparticles which have significantly reduced SAR and relaxivity upon PEGylation and on corona formation, attributed to reduced Brownian contributions and weaker NP solvent interactions. The PEGylated NF suspensions described here exhibit usable blood circulation times and promising retention of relaxivity in-vivo due to the strongly anchored PEG layer. This approach to biomaterials design addresses the challenge of maintaining magnetic efficiency of magnetic nanoparticles in-vivo for applications as theragnostic agents.

## 1. Introduction

The unique responses of magnetic iron oxide nanoparticles (MNPs) to applied magnetic fields combined with their biocompatibility and shelf-life, and the fact that the fields permeate tissue makes them attractive candidates for a range of biomedical applications [1]. In particular, dynamic magnetic alignment provides potential for theragnostic applications through the combination of three key responses. In strong homogeneous fields the moments align with the field altering the tissue ^1^H NMR relaxation times and generating contrast under different magnetic resonance imaging (MRI) modalities [2]. Secondly, in applied field gradients, translational forces provide potential for magnetophoretic MNP localisation [3], increasing accumulation at target sites [4]. While in alternating magnetic fields (>100 kHz) MNPs can act as potent heating mediators for thermally-triggered drug release and hyperthermic cancer treatment *e.g.* by localised ablation [5]. However, the theragnostic potential of MNP suspensions has not been fully realised due, in part, to the challenging surface chemistry strategies needed to stabilise the particles and retain their magnetic properties in biological media and complex environments.

Superparamagnetic MNPs produce heat on exposure to AC magnetic fields due to Néel (moment reorientation) and Brownian (particle diffusion) relaxation processes. With increasing particle size higher hyperthermic efficacies, quantified by the SAR values (W g^−1^ of Fe), are usually observed due to improved crystallinity (stronger moments as surface effects decrease with size) and optimal moment dynamics [6]. For spherical MNPs, on increasing core size to between 15–20 nm, the Néel contribution gradually decreases and the Brownian contribution comes to dominate [7]. *In vitro* and ex vivo studies [8,9], suggest that SAR is reduced due to loss of particle diffusion on immobilisation in tissue, or in the case of the circulatory system the bulky stabilizing ligands required to preserve colloidal stability in external magnetic fields. For instance, Liu et al. [10] reported that 19 nm spherical MNP suspensions lost up to 42% of SAR when the molecular weight of the PEG grafts was increased from 2 to 20 kDa. These effects are detrimental to *in vivo* applications of particles but suggest that heating mechanisms that are not primarily Brownian may be advantageous.

MNPs have also been shown to reduce the longitudinal and transverse ^1^H relaxation times of surrounding fluid and tissue, allowing them be used as efficient MRI contrast agents [2]. The ^1^H relaxation rate enhancements (per mM, Fe) for an MNP suspension as compared to pure water, *i.e.* the spin-lattice and spin-spin relaxivities, r_1_ and r_2_ respectively, are also determined by contributions from Néel and Brownian processes. The Brownian contribution to ^1^H relaxation is due to H_2_O (not particle) diffusion, and it dominates at the high fields (0.5–3.0 T, equivalent to ^1^H Larmor frequency, ν_L_, of 20–120 MHz) used for imaging. Factors which impact diffusion of H_2_O in the vicinity of MNPs magnetic moments, such as surface functionalisation with bulky biocompatible stabilizing ligands, are thought to reduce relaxivity in the MRI frequency range for single-core MNPs, although other factors are also likely to be in play.

While it may impact MNP properties, PEGylation has generally played a critical role in preparation of nano-probe surfaces for biological interaction, as it can extend blood circulation times [11] and modulate protein recognition and cellular uptake [12] PEG is believed to present a stable hydrogen-bonded hydration layer which, combined with configurational entropy arising from the flexible chains, inhibits protein adsorption [5]. Despite the hydrophilic nature of the polymer, significant reductions in both r_1_ and r_2_ of MNP suspensions on PEGylation have been reported. It has been suggested that these are due to increased distance of closest approach between H_2_O and the particle with increasing polymer molecular weight [13–15]. Relaxivity changes have also been ascribed to the nature of the ligand linker, or anchor; an r_2_ enhancement of 82% was reported for single core PEG grafted MNPs when conjugated π systems were present in the linker group [16]. However, as we have demonstrated [17] highly controlled experiments on fully dispersed suspensions are required to pinpoint such effects as r_2_, in particular, is highly sensitive to any MNP clustering. In many cases the causes of reduced relaxivity are unclear.

Multicore iron oxide nanoparticles, or nanoflowers (NFs), are *c.*22 nm structures assembled from partially-fused maghemite (γ-Fe_2_O_3_) grains to form flower-shaped nanoparticles. They have significant advantages over single core (usually spherical) equivalents; crystallographic continuity at the interfaces leads to cooperative behaviour that enhances susceptibility while maintaining superparamagnetic-like behaviour. NFs exhibit SAR values up to an order of magnitude higher than single-domain MNPs of similar size under same the AC-field conditions and useful r_2_ values [18]. In a detailed AC susceptibility and scattering study [19] it was demonstrated that, despite their relatively large size, the hyperthermic response of NFs is dominated by the Néel process in the therapeutic frequency range. This explains why the heating is relatively insensitive to changes in viscosity, which is an encouraging finding, but one that is yet to be fully exploited. The internal structure of NFs has been evaluated by HR-TEM complemented with magnetometry and other techniques including ferromagnetic resonance [20], and Mössbauer spectroscopy [21]. These studies convincingly show the presence of multiple exchange-coupled grains in each NF with some crystallographic continuity across the boundaries, although the organisation varies between different high-heating samples. For single core equivalents of similar size the moment dynamics are slow and the hyperthermic response weak [20].

Like their single core counterparts [22] NFs also require surface stabilisation with bulky hydrated ligands to provide necessary colloidal stability. Previous approaches to PEGylation of NFs included in-situ [23, 24] and post-synthesis ligand grafting [25–27]. However the impact of graft stability and chain length on the colloidal properties and different magnetic responses of NFs in buffers and biological media has not been fully evaluated. This information is needed to realise their potential for bio-application.

Here we describe a biomaterials design strategy that provides sufficient linker stability and PEG coverage to maintain full colloidal stability in field, enabling the first detailed evaluation of the impact of PEGylation and varying PEG chain length on the relaxivity and SAR of NF suspensions in complex media. We also examine the effects of π -conjugated linkers and of linker strength, while in all cases ensuring full particle dispersion. In contrast to previous reports for single-core MNPs of comparable size [10, 16, 13, 15], the relaxivity and SAR of aqueous dispersed NFs were shown to be unaffected by PEG chain length, up to 20 kDA, or by the linker group used. This is due to the predominant influence of internal magnetisation dynamics, as opposed to surface chemistry or graft dynamics. The resulting suspensions show long-term stability in buffer or complex media, with magnetic responses that are insensitive to particle concentration and biomolecule adsorption from complex media over many weeks.

To evaluate the clinical potential of NFs, an *in vivo* MRI study was carried out to confirm usable blood circulation and determine and uptake into a pancreatic cancer xenograft model, which was selected due to its challenging low permeability. PEGylated NF deposition was similar to that of liposomal formulations, providing a useful quantifiable MRI-measure of tissue-level liposome deposition. Critically, T_2_-weighted contrast was observed over several days post deposition. This demonstrates unusually good particle stability and retention of magnetic character in the tumour microenvironment, which we ascribe to the strong linker binding. PEGylated NFs are thus shown to have potential as theranostic tools that have long-term colloidal stability and good bio-distribution and imaging properties, which may also have sensitivity to AC-field stimulation for extended periods following deposition.

## 2. Materials and methods

### Synthesis of multi-core iron oxide nanoparticles (magnetic nanoflowers).

Multi-core maghemite, γ-Fe_2_O_3_, nanoparticles were synthesised using an adapted version of a previously reported polyol method [18]. Briefly, Iron (III) chloride hexahydrate (0.541 g, 2 mmol) and Iron(II) chloride tetrahydrate (0.199 g, 1 mmol) were added to 40 g of a liquid mixture of DEG and NMDEA 1:1 (v/v), in a three neck RBF. The solution was magnetically stirred overnight to ensure all material was completely dissolved. Separately, 0.32 g (8 mmol) of NaOH was crushed to a fine powder and added to 20 g of the DEG–NMDEA mixture and allowed to stir overnight to ensure full dissolution. The NaOH solution was then added to the solution of iron chlorides, and the resulting mixture was stirred for a further 3 h. The RBF was fitted with a Dimroth cooling condenser and was then clamped to a temperature-controlled heating mantle. The temperature was set to 220°C using the maximum heating ramp, once the target temperature was reached the solution was stirred for 12 h. The suspension was allowed to cool to room temperature, the black sediments were separated magnetically and washed with a mixture of ethanol and ethyl acetate (1:1, v/v) several times to eliminate organic and inorganic impurities. Possible iron hydroxides were removed by rinsing the particles with 10% nitric acid. 4.13 g of iron(III) nitrate was then dissolved in 10 mL of water and added to the nanoparticles. The resulting suspension was heated to 80°C for 45 min to achieve complete oxidation of the nanoparticles. After another treatment with 10% nitric acid, the particles were washed twice with acetone and diethyl ether. The particles were then dried under a gentle flow of N_2_ and redispersed in 10 mL of water.

### PEGylation of magnetic nanoflowers.

PEG-Gallol was synthesised using a modified version of the synthesis described by Guardia et al. [28] 4 g of PEG (average MW 8 kDa) was dissolved in 70 mL of THF. 0.086 g of gallic acid and 0.0089 g of DMAP was then added to the solution. The solution was stirred at room temperature until full dissolution was achieved. 0.5256 g of DCC in 5 mL of THF was then added dropwise. The solution was stirred at room temperature for 16 h. THF was then removed using a rotary evaporator. 200 mL of water was then added, and the pH of the solution was adjusted to pH 2 using 1 M HCl. Unreacted PEG precipitated out at this point. The solution was vacuum filtered and the filtrate, containing the product, was isolated. The product was extracted four times into CHCl_3_ and recovered on a rotavap. The recovered product is believed to consist predominately PEG-Gallol, although unreacted PEG may still be present. As unreacted PEG will not bind to the particles, further clean-up is not required at this point as free PEG can be removed from the final PEGylated particle suspensions using dialysis. An aqueous stock solution of the polymer was then prepared at pH 7, 0.02 mol of PEG-gallol was then added to the BNF suspension per 1 mol of elemental iron. The suspension was then placed on a shaker for 30 min to complete the reaction.

### AC-field hyperthermia measurements.

AC hyperthermia analysis was performed using a NanoTherics Magnetherm NAN201003 (NanoTherics Ltd, UK) hyperthermia measurement system. The system allows measurement of temperature vs time via a non-metallic OP-Sens optical thermometer to avoid eddy currents. The system can apply an AC magnetic field at frequencies from 100 to 1000 kHz. 1 mL of the aqueous solution was transferred into a pre-weighed Eppendorf tube. The optical thermometer was submerged in the aqueous suspension via a hole in the Eppendorf cap. The sample was placed in a thermally insulating polystyrene sample holder to maintain adiabatic conditions. Samples were run at room temperature, with a field strength of 15.9 kA m^−1^ and an AC frequency of 530 kHz.

### Fast field cycling NMR measurements.

Relaxation properties of nanoparticle suspensions were measured between the fields of 0.01 to 40 MHz using a Stelar Spinmaster FFC-2000 Fast Field Cycling NMR Relaxometer. The RF pulses were controlled using a Spinmaster RF Console (Stelar, Mede, Italy). The temperature was controlled using a Spinmaster Variable Temperature Controller (Stelar, Mede, Italy). The temperature was set to 25 ± 0.1°C, unless otherwise stated, and allowed to thermally equilibrate for 10 min prior to measurement. Two pulse sequences were used to measure T_1_, for frequencies higher than 12 MHz a non-polarised (NP) sequence was used while for frequencies below 12 MHz a pre-polarised (PP) sequence was used. T_2_ measurements were carried out on a Maran Ultra 23.4 MHz NMR Analyser (Oxford Instruments, United Kingdom) at 25.0 ± 0.1°C, using a Carr-Purcell-Meiboom-Gill sequence (CPMG/S).

The ^1^H relaxation enhancements due to suspended particles are quantified by the spin-lattice and spin-spin relaxivities, r_1_ and r_2_:

(1) $$r_{1/2}\;=\;\frac{{\text{R}}_{\text{1/2 (meas)}}-{\text{ R}}_{\text{1/2 (solvent)}}}{\left[\text{Fe}\right]}\;$$

where r_1/2_ have units s^−1^ mM^−1^, R_1/2(meas)_ and R_1/2(solvent)_ are the measured relaxation rates of the suspensions and of the particle-free solvent, respectively.

Average r_2(*in vivo*)_ values at can be estimated using the Fe concentration, as determined by ICP following:

(2) $$r_{2\left(in\;vivo\right)}\;=\;\frac{△R_{2}}{{\left[Fe\right]}_{ICP}}\;$$

### Cell culture and cytotoxicity studies.

HK-2 cells (immortalised human renal proximal tubular epithelial line) were maintained in Dulbecco’s Modified Eagle’s Medium (DMEM)/Nutrient Mixture F-12 Ham with 15 mM HEPES and sodium bicarbonate (Sigma-Aldrich), supplemented with 10 ng mL^−1^ human recombinant epidermal growth factor (hEGF), insulin-transferrin-selenium (ITS) at 5 μg mL^−1^ and 5 ng mL^−1^, respectively, 36 ng mL^−1^ hydrocortisone, 3 pg mL^−1^ tridothyronine (all Sigma-Aldrich), 2 mM L-glutamine (Invitrogen), 100 μg mL^−1^ penicillin and 100 μg mL^−1^ streptomycin (Gibco). Cells were maintained at 37°C in a 95% air/5% CO_2_ environment and growth medium was replenished every 48 h. Cells typically became confluent within 4 days and were sub-cultured using 0.05% trypsin-EDTA (Gibco). DMEM/Nutrient Mixture F-12 Ham with 15 mM HEPES and sodium bicarbonate, supplemented with 10% Foetal Bovine Serum (Gibco) was used to neutralise trypsin during passaging. The 3-(4,5-dimethylthiazol-2-yl)-2,5-diphenyltetrazolium bromide (MTT) colorimetric assay was used to assess gross cytotoxicity of P_8K_NF-8K *in vitro*. HK-2 cells were seeded at a density of 7.5×10^3^ cells in 96-well culture plates. Cells were incubated at 37°C in a 95% air/5% CO_2_ environment for 24 h and were then treated with varying concentrations of P_8K_NF diluted in HK-2 culture medium. After 48 h, medium containing PEGylated NFs was aspirated and MTT solution (Sigma-Aldrich) diluted in HK-2 culture medium at a final concentration of 0.5 mg mL^−1^ was added to wells. Plates were incubated for 3 h at 37°C in a 95% air/5% CO_2_ incubator. Culture medium containing MTT solution was then aspirated and 100 μL dimethyl sulfoxide (DMSO) (Sigma-Aldrich) was added to dissolve the formazan crystals generated. Absorption of wells was measured using a microplate reader (CLARIOstar, BMG Labtech) at 570 nm. Cell viability was subsequently calculated.

### *In Vitro/ Vivo* MRI measurements.

Tumours derived from a patient derived xenograft (PDX) PDAC tumour model (#18269) were used as *in vivo* models. The PDX model was developed at Roswell Park Comprehensive Cancer Centre [29]. Small tumour fragments (c.8 mm^3^) from donor mice were implanted subcutaneously on the abdominal wall of anesthetised 18 to 20 g CB17 SCID mice. Once tumours reached a volume greater than 200 mm^3^, mice were randomised into groups and studies were initiated. All procedures were approved in advance by the Institutional Animal Care and Use Committees of Roswell Park and the University at Buffalo, State University of New York. For standard MRI analysis, n = 3 mice were treated with particle formulations (FMX or P_8K_NFs) in 10% dextrose at a dose of 50 mg kg^−1^ (mg of Fe per mass of mouse) using tail vein injections. FMX (Feraheme^®^) was purchased from AMAG Pharmaceuticals, USA. PNFs with a PEG-Gallol chain length of 8 kDa were used in all biological studies.

MR imaging was undertaken on a 4.7 T preclinical MR imager utilising the ParaVision 3.0.2 imaging platform with a 35 mm I.D. radiofrequency transceiver coil (Bruker Biospin, Billerica, MA). Air temperature within the bore was maintained at 37°C and samples were allowed to equilibrate thermally for 15 min prior to measurements. T_2_ relaxation rates (to evaluate r_2(*in vitro*)_) were acquired using a CPMG spin-echo sequence with a fixed TR of 3000 ms, and measurements were carried out with varying TE, ranging from 20–1200 ms in 20 ms increments. Using Analyze 7.0 software (Analyze Direct, Overland Park, KS), regions of interest (ROIs) were drawn for recorded datasets and signal intensities were extracted using routines developed in house with MATLAB (MathWorks, Natick, MA) [30]. R_2_ was determined by fitting signal intensity against TE with an mono-exponential decay in GraphPad (GraphPad Software, San Diego, CA).

ICP analysis was undertaken to determine Fe concentration of tumours following FMX and PNF treatments, employing a modification of the method reported by Dong et al. [31]. To ensure homogeneity of the samples, tumours were ground while frozen and a small portion was weighed and placed in 15 mL centrifuge tubes. 1 mL of 12 M HCl and 3 mL of 16 M HNO_3_ were added and the samples were allowed to sit for 1 hour on the bench. Following this, the samples were placed in an oven at 60°C for 2 h before cooling to room temperature. Appropriate dilutions were carried out with 1 M HNO_3_ and samples were submitted for ICP analysis to the National Centre for Isotope Geochemistry, UCD. Measurements were carried out on the iCAP-Q ICP-MS (ThermoFisher) in high matrix and collision cell mode, using He as the collision cell gas (4.85 mL min^−1^). Samples were introduced into the mass spectrometer through a cyclonic, Peltier-cooled spray chamber with an ESI PFAST nebuliser at a rate of 100 μL min^−1^ and ^56^Fe and ^57^Fe were monitored. Washes between samples consisted of 80 seconds of 5% HNO_3_ with accelerated peristaltic pump speed, followed by 80 seconds of sample take up. For quantification, external standardisation with dilutions of a Sigma-Aldrich TraceCert periodic table mix 1 (Lot #BCBR7889V) was used for a concentration range between 1 and 400 ppb with sample standardisation carried out every 20 to 30 samples to account for instrumental drift. Data was processed using the QTegra software package (ThermoFisher).

Fluorescent sterically stabilised liposomes (F-SSL) were prepared, as previously reported [32], by a thin film hydration method, at a total lipidic concentration of 20 mM using DSPC, DSPE-PEG-methoxy and cholesterol in a 9:1:5 molar ratio including 0.01 mol% of the fluorescent, nonexchangeable dialkyl carbocyanine membrane labels DiR-DS. The lipids, dissolved in CHCl_3_, were mixed together and the CHCl_3_ was removed slowly on a rotavap to form a thin lipidic film. The film was resuspended in a desired volume (typically 5 mL) of preheated 45°C 25 mM HEPES buffer (with 140 mM NaCl and pH value of *c.*7) with vigorous shaking in water bath and vortexing. The resulting solution was sonicated for 4×30 second intervals with intermittent shaking in water bath and vortexing. The liposome solutions were immediately extruded through polycarbonate filters to provide vesicles in the required size range. Liposome diameter was measured by dynamic light scattering (NanoBrook Omni Analyser, Brookhaven Instruments, Holtsville, NY). F-SSL (1 μmole/animal) were injected IV at intervals following initiation of treatment to probe vascular permeability and perfusion. Animals were sacrificed after 24h at the time of peak SSL deposition. The tumours were rapidly frozen and serial sections were obtained for analysis. Unfixed sections were mounted with DAPI-containing anti-fade mounting medium. Panoramic images of F-DiI deposition and lectin-FITC were acquired that captured the whole tumour section using a Leica DM8 system with a 20X/0.8 objective and standard Cy5 filter, with identical exposure conditions to allow comparison among individual tumours. Fluorescent intensity in all tissues was quantified using Fiji (NIH).

Dynamic contrast-enhanced measurements (DCE-MRI) were obtained with Gd-DTPA as the contrast agent (300 μmole kg^−1^). T_1_ relaxation rates were determined using an inversion-recovery True-FISP acquisition with the following parameters: TE/TR=1.5/3.0 ms, flip angle=30°, inv. repetition time=10 s, segments=8, frames=100. R_1_ measurements were acquired serially prior to, and for 30 min after Gd-DTPA administration. Signal intensities were extracted from each ROI, and T_1_ relaxation rates were calculated using routines developed in-house with MATLAB. The concentration of Gd-DTPA ([C]_*t*_) at time *t* was calculated as:

$$C_{t}\;=\;\frac{R_{1\left(t\right)\;}-R_{1\left(0\right)}\;}{r_{1}}$$

where *R*_1(*t*)_ is the T_1_ relaxation rate at *t, R*_1(0)_ is the baseline relaxation rate, and r_1_ is the T_1_ relaxivity of Gd-DTPA, taken as 4.0 mM^−1^ s^−1^. The area-under-the-curve (AUC) of Gd-DTPA deposition in the tumour ROI is reported, and was derived in MATLAB.

### Dynamic light scattering measurements.

Dynamic light scattering (DLS) measurements were performed using a Malvern Nano ZetaSizer (Malvern Instruments, Malvern UK). Data was processed using Dispersion technology software (v 7.13, Malvern instruments, Malvern UK) using the multiple narrow modes algorithm based upon a non-negative least square fit to calculate the hydrodynamic diameter and the PolyDispersity Index (PDI). The z-average is an intensity-based overall average hydrodynamic size based on a specific fit to the raw correlation function data. The PDI is a measure of the degree of polydispersity of the sample. Zeta potential measurements were performed which measure the surface charge of particles by measuring their velocity while they are moving due to electrophoresis. Samples were diluted to c.5 mM and analysis was performed at 25°C. All measurements were performed three times, the average with standard deviation was reported.

### Transmission electron microscopy.

TEM was performed on a FEI Tecai G2 TWIN 200 kV microscope. Aqueous suspensions of NFs were prepared (concentrations of c. 5 mM Fe) and 5 μL was pipetted onto a carbon TEM grid (Formvar and carbon films on 400 μm copper grid, Agra Scientific).

### Statistical Analysis.

The size of each iron oxide core was determined as the average of the longest and shortest identifiable axes from the TEM images. The average iron oxide core size, d_TEM_, of BNFs was calculated by averaging this size for at least 250 nanoparticles. The data was fitted to a lognormal distribution using OriginPro 8.5, for illustrative comparison with dynamic light scattering data of the same BNF sample.

Statistical analysis for the *in vitro/vivo* MRI analysis was undertaken using two-tailed unpaired t-tests at a 95% confidence interval. Where relevant, statistical analysis is noted in figures and tables with ^∗^ indicating P<0.0332, ^∗∗^ indicating P<0.0021, ^∗∗∗^ indicating P<0.0002 and ^∗∗∗∗^ indicating P<0.0001.

## 3. Results and discussion

### 3.1. Synthesis and characterisation of magnetic nanoflowers

An adapted polyol synthesis [18] was used to prepare multiple batches of stable aqueous suspensions of monodisperse multicore iron oxide nanoflowers. The physical and magnetic properties of the products from 10 standard syntheses were fully characterised, Fig, 1a, b demonstrating that the approach is highly reproducible. The bare or uncoated nanoflowers will be referred to as BNFs, and the PEGylated equivalents as PNFs. TEM analysis for BNFs confirms the flower-like morphology, with multiple grains apparent. The inorganic core size d_TEM_ was 22.5±2.3 nm (population average for all batches), Fig, 1c, d. The forced oxidation step is known to predominantly provide the expected maghemite, γ-Fe_2_O_3_, phase [20] *i.e.* any Fe_3_O_4_ formed is fully oxidised. This conclusion is supported by previous XRD analysis [20], and by the fact that for BNFs extending the oxidation time, or aging the suspensions for months had no effect on the magnetic properties. In addition room temperature DC-magnetometry, Fig. S1, confirms high saturation magnetisation, M_s_, and magnetic susceptibility, χ, as expected for NFs.

**Fig. 2.**
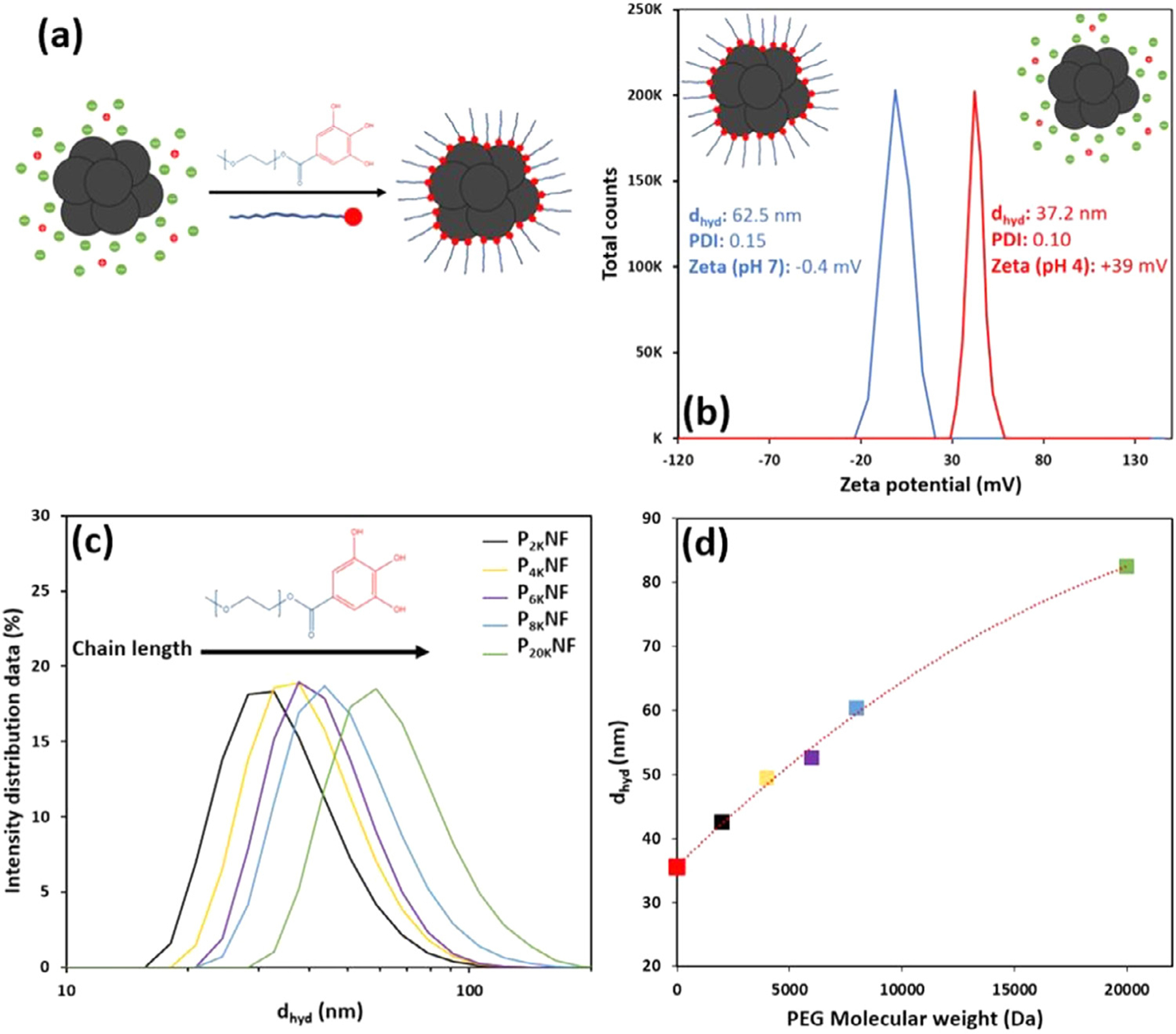
Characterisation of PEGylated nanoflower (PNF) suspensions. (a) Schematic representation of the PEGylation of nanoflowers, illustrating the transition from electrostatic to steric stabilisation. (b) Zeta potential distributions for BNF (red) and P_8K_ NF (blue) suspensions at pH 4 and 7 respectively, showing a change from significantly positive to near neutral surface charge with full dispersion retained. (c) Size distribution by intensity for aqueous PNF suspensions with varying chain length. (d) Dependence of average d_hyd_ on PEG molecular weight for the same suspensions at neutral pH, the value for a BNF suspension (at pH 4, 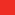) is also included.

Colloidal stability of aqueous BNF suspension is observed for 5 ≥ pH ≥ 10, arising from electrostatic repulsion driven by the charge of surface oxide, Fig. 2b and S2. Low and high pH BNF suspensions from different preparations consistently exhibit low z-average hydrodynamic size, d_hyd_ *c.*35 nm, with good monodispersity, PDI<0.13, and these values are unchanged over at least 6 months. The isoelectric point of the BNF suspensions was estimated as pH 7.2, Fig. S2. As expected, under neutral conditions, or in chemically complex or biological environments, electrostatic stabilisation does not maintain BNF colloidal stability/dispersion, Fig. S2. This sedimentation can be accelerated by application of strong magnetic fields, such as those used in MRI.

The batch-to-batch reproducibility of BNF size provides consistency in the hyperthermic efficacy, measured by the SAR value (at 530 kHz, 15.9 kA m^−1^ see Experimental), of 300±22 W g^−1^ corresponding to an intrinsic loss power, ILP, of 2.2 nH m^2^ kg_Fe_^−1^. These values are independent of concentration (over the range 5 to 100 mM, or 0.28 to 5.6 mg mL^−1^, Fe) and of the position within the sample at which the temperature is measured, demonstrating colloidal stability (no measurable sedimentation or aggregation) in the high Fe concentration range used for hyperthermia. Higher SAR values have been reported for NFs [18], however to our knowledge detailed reproducibility and robustness has not been. Having established reproducibility for the BNF preparation, with SAR variation ≤ 10%, it is possible to evaluate the effect of PEGylation and to provide consistent materials for *in vivo* analysis, for which multiple batches are required.

### 3.2. PEGylation of magnetic nanoflowers

Polyethylene glycol grafting was selected to provide a steric barrier to attractive interparticle forces, Fig, 2a, with a view to enhancing colloidal stability in complex media, reducing protein recognition and extending blood circulation times. The trihydroxybenzene-derived functionalised polymer, referred to here as PEG-gallol, was selected as we have demonstrated it provides chemically stable links to spherical MNPs [33] through a facile single-step. The process is effectively quantitative, with convenient purification (see Experimental). Stable suspensions of PEGylated nanoflowers (PNFs) were prepared with PEG MW ranging from 2–20 kDa. Ligand binding (formation of a strong bond to FeO) is further demonstrated by modification of the PEG decomposition temperature revealed by TGA analysis, Fig. S3. TEM analysis demonstrates no significant change in PNF size, d_TEM_ 21.4±1.8 nm, or morphology as expected. The impact of PEGylation on the physical properties of the aqueous suspensions of different chain lengths is summarised in Fig, 2.

PNFs, unlike BNFs, show full dispersion and colloidal stability at neutral pH despite negligible surface charge. For P_8K_NFs (8 kDa grafts) at pH 7 typical values of d_hyd_ 63 nm, PDI 0.15 and zeta potential (z_p_) −0.4±2 mV, Fig, 2b, were measured. The d_hyd_ and PDI were almost unchanged on decreasing the pH to 4, confirming steric stabilisation of PNFs. This is unlike BNF suspensions for which stability requires pH that supports a high z_p_ (*e.g.* +39±2 mV at pH 4, Fig, 2b). Stable PNF suspensions were formed up to 20 kDa PEG, the highest molecular weight studied. Significant increases in d_hyd_ were measured on PEGylation, Fig, 2c, d, with size progressively increasing from 43 to82 nm with increasing PEG chain length. Monodispersity was retained in all cases, with PDI values increasing from 0.12 (P_2K_NFs) to 0.15 (P_20K_NFs). Our interpretation, which is confirmed by the retention of magnetic properties in suspension shown below, is that for all chain lengths the particles are fully dispersed, *i.e.* the increase in d_hyd_ (from 35 nm for BNFs) arises due to longer grafts only, and not from any aggregation. The strength of the FeO-gallol linkage affords chemical stability and as a result the suspensions are also stable in buffers, see below. This advantage combined with the possibility of varying chain length may be useful for in-vivo applications; it has been reported that graft chain length, which determines the hydrodynamic size, plays a significant role in extending circulation times of spherical MNPs [11,34]. We note that the PEGylation approach may also be used in conjunction with other optimised polyol NF syntheses [35], which show very promising improvements in SAR.

### 3.3. Impact of PEGylation on the magnetic properties of nanoflowers in suspension

**Fig. 3.**
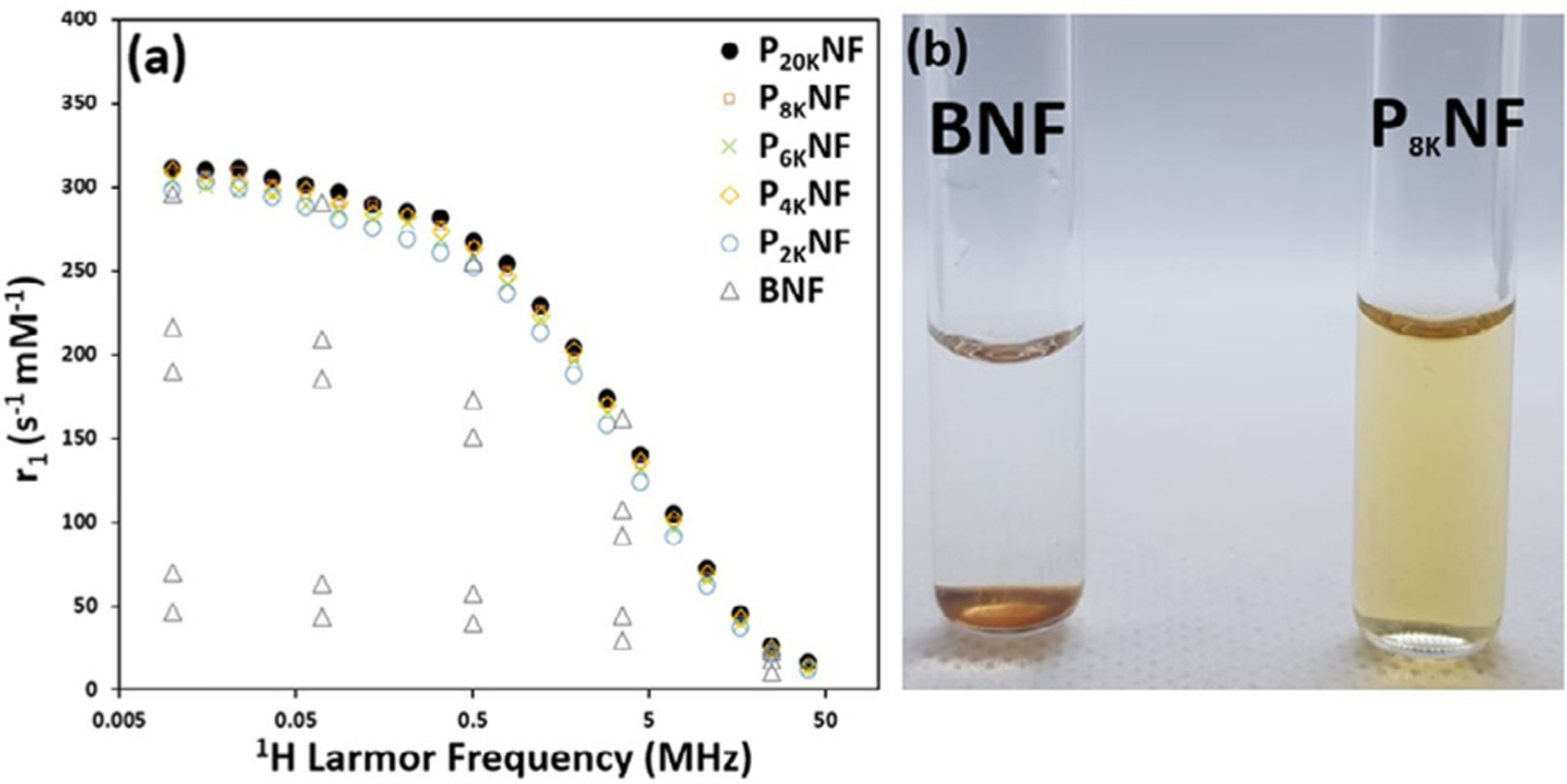
(a) FFC-NMR profiles (^1^H Larmor frequency dependence of the spin-lattice relaxivity) recorded in H_2_ O at 25 °C for PNF suspensions with varying graft chain lengths. Also included are apparent-r_1_ values (∆) for successively recorded profiles for a BNF suspension. The gradually decreasing values demonstrate field-dependent instability of the non-PEGylated suspensions. (b) Images of BNF and P_8K_ NF suspensions taken 12 h post FFC-NMR analysis.

The impact of PEGylation on both the magnetic properties and the colloidal stability of NF suspensions in the presence of magnetic fields was examined. Fast field cycling NMR relaxometry was used to measure the H_2_O spin-lattice relaxivity, r_1_, of BNF and PNF suspensions in the ^1^H ν_L_ range of 0.01–40 MHz (equivalent to 0.25 mT-1.0 T), *i.e.* the FFC-NMR profile. This technique is established for providing insight into MNP suspensions as the profile shape is highly sensitive to the local particle magnetic ordering, as sensed by the diffusing solvent molecules [36]. The high frequency r_1_ values, which are into the clinical MRI range, measure the MRI contrast generation efficacy (under T_1_-weighted conditions) *in vitro*. Typical FFC-NMR profiles of aqueous NF suspensions are presented in Fig. 3.

The profile shapes of BNF and PNF suspensions, Fig. 3a, appear similar and they are very like those previously reported for citrate-stabilised NFs [20], but with slightly reduced r_1_ values. Super-imposibility of the profiles shows that the Néel dynamics of the moments, which dominates ^1^H relaxation in the low frequency range (see next section), is not altered on increasing the chain length. Hence the increases in d_hyd_ with chain length, Fig, 2d, are not due to aggregation which would slow Néel reorientation. It is clear that once the particles are fully dispersed surface chemistry, and indeed PEG chain length, have no impact on relaxivity. This result highlights the superior utility of multicore nanoparticles for in-vivo MRI applications.

For all chain lengths PNF suspensions were shown to be stable to pH (unchanging d_hyd_ and PDI) on exposure to fields of up to 1.4 T for many hours, which is critical for application. Interestingly, for BNFs only, repeating the FFC-NMR measurement resulted in a decrease in relaxivity which, as shown, progressed for each successive measurement. So for BNFs we refer to these as apparent-r_1_ values. This change arises due to gradual ongoing aggregation and rapid sedimentation of the aggregates. As the relaxivities are normalised by the initial Fe concentration the apparent-r_1_ values decrease as the number of BNFs in free suspension falls, but the frequency dependence does not change significantly. Complete sedimentation of the BNF suspension is observed 12 h after the initial exposure to 0.5 T (40 MHz), Fig. 3b. Sedimentation was also an issue for the citrate-stabilised NF suspensions previously reported [20], although in that case the aggregation/sedimentation was slower than for BNFs and the profiles could be reliably recorded. Field instability, and the necessity of isotonic media, render electrostatically stabilised NF suspensions of little value for *in vivo* MRI, emphasizing the need for PEGylation.

**Fig. 4.**
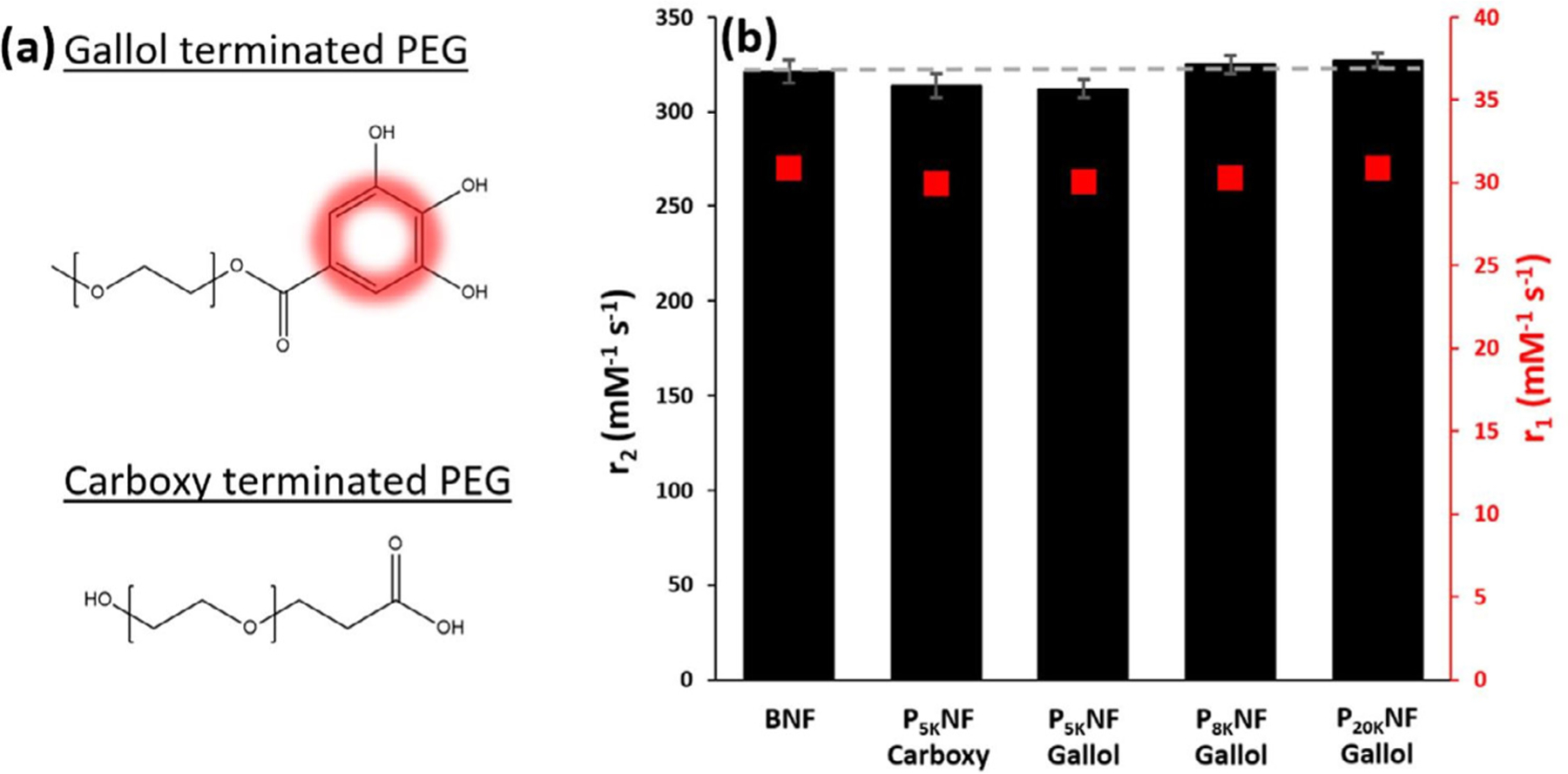
(a) Illustration of the molecular structure of gallol and carboxy terminated PEG. (b) ^1^H longitudinal (r_1_ ) and transverse (r_2_ ) relaxivity values, recorded at 25 °C and 24 MHz, for BNF and PNF H_2_O suspensions stabilised with different PEG chain length and PEG linker groups.

The impact of the surface layer on relaxivity was further examined by changing the PEG linking group. PNF suspensions stabilised with PEG 5 kDa were prepared using gallol- and carboxy-terminated polymers. Despite the change in linker, the hydrodynamic sizes and colloidal stability were again very similar, with d_hyd_ 54 nm (PDI 0.10), and 58 nm (0.09) obtained respectively. DLS intensity distributions are overlayed in Fig. S4. In this case r_2_ measurements (at 24 MHz) were used both because the spin-spin relaxivity is highly sensitive to any aggregation and to evaluate the contrast potential under T_2_-weighting MRI conditions. It was found that the values were not affected by either the linker group used or the PEG chain length, Fig. 4. The average r_2_ value for all suspensions shown of, 320 mM^−1^ s^−1^ ± 1.9 %, is comparable to literature values for multicore particles of similar size at fields below 1 T [20, 37]. The r_1_ values were also unaffected and the similar ratios, r_2_/r_1_, again demonstrate that all the suspensions are fully dispersed (no aggregation). Together the high r_2_ values, field stability and low d_hyd_ suggest PNFs may have utility as contrast agents in T_2_-weighted MRI, this potential is evaluated below. This observation is in contrast to a widely cited study by Zeng et al. [16], which reported an r_2_ enhancement of 82% in single core PEGylated MNPs when a conjugated catechol linker group was used instead of an unconjugated diphosphate group. The observed increase in r_2_ was attributed to the π –π conjugation in the catechol linker increasing the inhomogeneity of the local magnetic field around the NP.

### 3.4. MRI relaxivity mechanisms in nanoflower suspensions

Optimisation of relaxivity is key for the development of improved MRI contrast agents [38]. However deeper understanding of r_1_ and r_2_ is complicated by their involved dependence on particle size, magnetisation, hydration, and moment and solvent dynamics, which is described for NFs in this section. In addition to evaluating contrast potential in the clinical range FFC-NMR profiles provide insight into how these different factors contribute to the magnetic order of suspended MNPs. The high frequency part of the profile is dominated by Brownian relaxation, *i.e.* modulation of the ^1^H-particle moment dipolar interaction by water diffusion, and for NF suspensions is similar in appearance, Fig. 3a, to that observed for superparamagnetic (SPM) MNPs [38]; with r_1_ strongly decreasing with increasing frequency. The low frequency response is markedly different; with a monotonous increase of r_1_ with decreasing frequency observed, instead of the characteristic SPM mid-frequency r_1_ maximum and low frequency plateau [38]. As noted above, in this frequency range the moments are not locked to the weak external field and the Néel process dominates; relaxation is driven by modulation of the ^1^H-moment dipolar interaction arising from moment dynamics within the local magneto-crystalline field. For NFs inter-grain exchange coupling strongly dampens Néel dynamics, as compared to the SPM case, resulting in higher r_1_ values. Similar behaviour has been observed for DNA stabilised MNP clusters where interparticle interactions (dipolar in that case) are also strong [39].

The fact that PNF relaxivities and profile shapes are independent of PEG chain length demonstrates the absence of any chain length dependence of; (i) water diffusion past the particle, which would alter the Brownian correlation time, τ_B_ (that determines the high frequency part of the profile); (ii) average distance of closest approach for H_2_O, which would alter r_1_ at all frequencies; (iii) particle aggregation in the field, which, as noted above, would alter the Néel correlation time, τ_N_, that determines the low frequency part. At MRI concentrations PEG (in this chain length range) effectively prevents field-induced aggregation, which is often observed in strong magnetic fields even for surface-functionalised NFs [27], and the intrinsic magnetic character of the dispersions are therefore identical.

We recently prepared spherical 8–9 nm superparamagnetic MNPs under identical conditions and bound bi-functional silane linkers to the surfaces (with the silane function linked to FeO) eliminating any differences due to the linker group to the oxide, which were then used to graft either glycopeptides or jeffamine. It was observed that once the d_hyd_ and PDI values were the same (*i.e.* the particles were fully dispersed) the profiles were superimposable [17]. The results shown here for NFs demonstrate that once full dispersion is achieved relaxivity is independent of chain length or the nature of the hydrated polymer stabiliser for even larger particles (d_TEM_ 22.5 nm for NFs), and for complex shapes.

For the remainder of this sub-section the details of the effect of linker group on relaxivity are discussed in the context of the literature. Fig. 4 demonstrates no dependence of the r_1_ or r_2_ values on the linker binding strength be it strong (gallol) or moderate (carboxy) for PNF suspensions, once the d_hyd_ and PDI values are controlled, Fig. S4. In an article, noted above), [16] unusually high r2 values were noted for small (d_core_ 3.6 nm) PEG stabilised spherical MNP suspensions when relatively weakly binding hydroxamate linkers were used. The authors attempted to separate the effects of binding group strength and coverage on r_1_ and r_2_ using the accepted model, see below. Many previous studies have attempted to rationalise improvements in relaxivity for MNP suspensions in terms of anticipated effects of the ligand binding strength [16,40] and hydration [13,41–43] using the model [38,44].

In the accepted model the spin-spin relaxivity, r_2_, for instance, is shown to be dependent on M_s_, d_core_ and L, the thickness of the water impermeable layer. The particles are taken to be perfectly monodisperse and sharp spherical boundaries are assumed corresponding to transitions from crystalline to canted oxide within the cores, at the core surface, and at the solvent-accessible surface. In reality any such boundaries are on the molecular scale, so variations in local geometry are inevitable and surface texture may also play a role. Nevertheless, application of the model can be instructive [45], and changes in relaxivity have been attributed to the type, length or coverage of grafted polymers. However sometimes the hydrodynamic size and monodispersity are poorly controlled or not reported. In the report describing hydroxamate linkers [16] increases in ligand binding strength were shown to significantly reduce the measured M_s_ (thicker canted oxide layer, as expected), and increases in coverage were suggested to decrease τ_D_. Close examination of the data shows that the expected Ms^2^ dependence is not observed for MNPs (with catechol and hydroxamate linkers) of almost identical coverage for the same molecular weight polymer. Critically the d_hyd_ and PDI values of the suspensions were not provided, so aggregation may play a part. The possibility of using linker binding strength to improve relaxivity for smaller particles remains plausible as the fraction of the oxide in the surface canted layer does become significant for smaller particles and, as was demonstrated, this aspect is sensitive to the ligand head-group [16]. It is likely however that weaker binding, to which high r_2_ was attributed in this case, is likely to compromise stability in biological media.

In summary we show that for NFs linker binding strength effects are not observed. This may be because the thickness of any canted layer is less important (larger particle size), or higher local curvature (than for spherical MNPs) may dominate over any ligand-mediated effects. In any case, the advantages of NFs for T_2_-weighted MR imaging are that strongly bound polymers can be used to provide colloidal stability in complex media (not the case for hydroxamate linkers) and the magnetic properties are determined by inter-grain as opposed to surface interactions and so are less sensitive to the environment. These potential advantages are explored for MRI and AC-field hyperthermia applications below.

### 3.5. AC-field hyperthermia mediation in suspension

In AC-field hyperthermia studies, for which the goals are usually to achieve bulk temperature increases [46] and/or to determine SAR values, the concentrations used are typically an order of magnitude higher than for magnetic resonance. The impact of PEGylation and polymer chain length on the AC-field magnetic heating response of NFs in suspension over a wide concentration range (10–50 mM) was therefore evaluated, Fig, 5. PEGylation, in the chain length range 2 to 20 kDa, was found to have minimal impact on the magnetothermal response. The SAR of the PNF suspensions (294 W g^−1^ ± 5.3, averaged for all concentrations and chain lengths) was within 2% of the BNF value, *i.e.* within measurement error, the full data is shown in Fig. S5. These observations suggest minimal aggregation or dipolar interactions between particles, as was the case for the relaxivity, extending the concentration range within which the intrinsic magnetic character of the dispersions remain unchanged up to 50 mM Fe. They also show minimal effect of PEGylation on the magnetic properties of the dispersed particles. That is in contrast to studies which describe significant, 42%, SAR loss for similarly sized (19 nm) single core particles on increasing the MW of the grafted PEG from 2 to 20 kDa [10], observations which were attributed to the bulky adlayer hindering the Brownian dominated heating mechanism for > 15 nm particles. Our results illustrate the advantage of utilizing particles with Néel-dominated hyperthermic responses, which retain heating efficiency even with high molecular weight PEG grafts, once full dispersion is ensured. Finally, PNF suspensions also show a linear dependence of SAR on AC-field strength, Fig. S5, down into the range that can be applied clinically [47].

### 3.6 In vivo *MRI analysis of P*_*8K*_ *NF suspensions*

#### 3.6.1. Stability in complex media

**Fig. 6.**
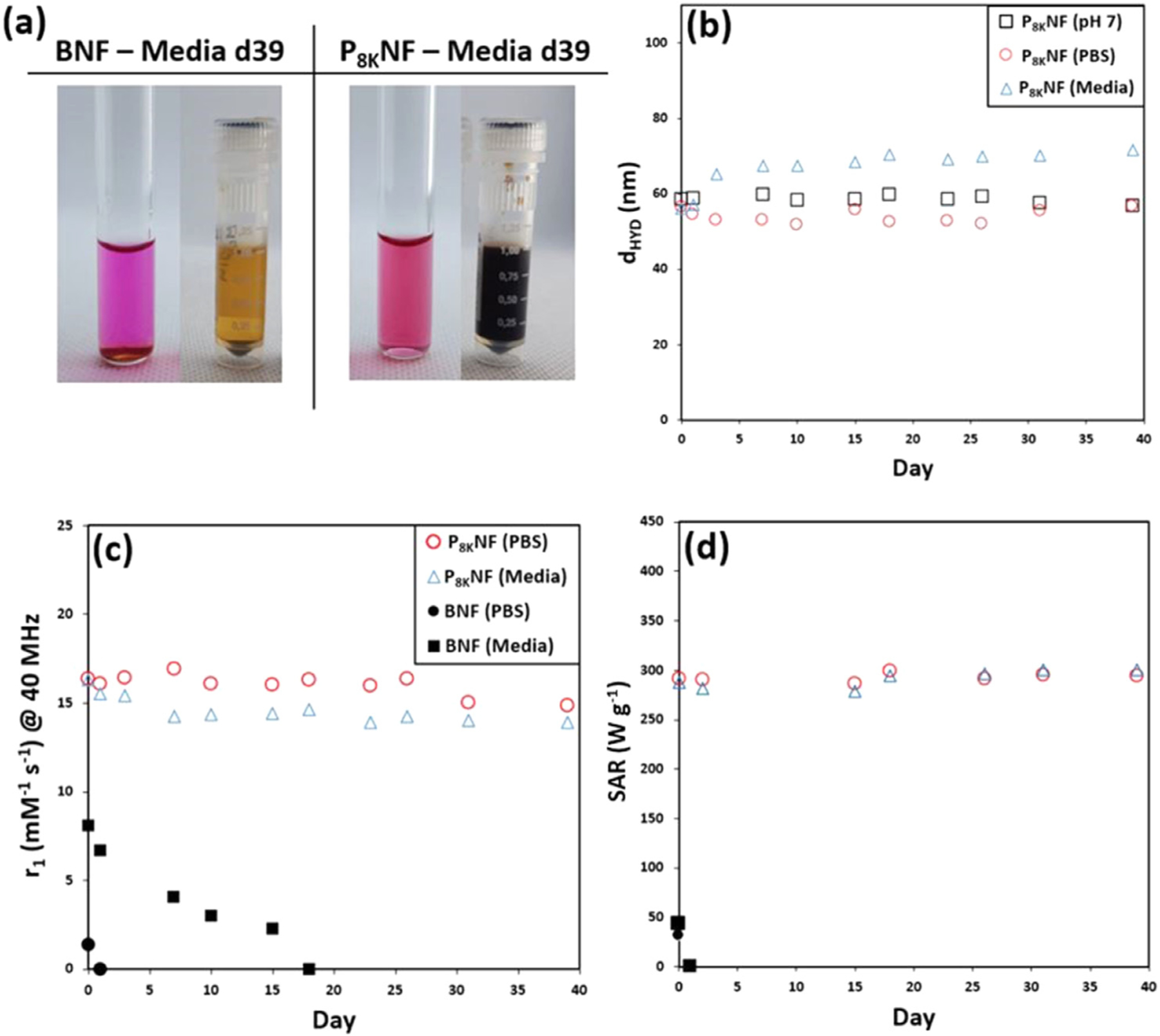
(a) Images of BNF and P_8K_ NF suspensions in media at low (left) and high (right) Fe concentration after 39 days. (b) Plot of the hydrodynamic size of P_8K_NF suspensions in different dispersants over 39 days. (c) Analysis of the relaxivity, and (d) SAR values of BNFs and P_8K_NFs in different dispersants over 39 days.

Prior to bio-studies the long-term stability of P_8K_ NF in biologically relevant dispersants was evaluated. P_8K_ NF was selected as it represents an intermediate polymer shell thickness within the investigated range. Phosphate buffered saline (0.1 M PBS) and a representative cell culture media cocktail were selected. The latter comprised Dulbecco’s modified Eagle’s medium (DMEM) and Fetal bovine serum (FBS) and contains over 1000 components, many of which have the potential to compromise colloidal stability. The colloidal and magnetic properties of these PNF suspensions were analysed at 50 and 1 mM Fe, concentrations appropriate to hyperthermia and MRI applications, respectively, and their performance over 39 days was compared with BNF suspensions, Fig. 6.

At both concentrations the BNF suspensions rapidly aggregated and sedimented (pellet visible in both cases). On the other hand PNF suspensions exhibited good long-term colloidal stability in both PBS and cell culture media over a 39-day period and, as noted above, to repeated external magnetic field exposure. The colloidal and magnetic properties of the PNF suspensions over the course of the stability study are summarised in Table 1. In PBS suspension P_8K_NF were stable over 39 days, with no substantial change in hydrodynamic size or PDI observed, Fig. 6a; >90% of relaxivity was retained, Fig. 6c, and no loss of SAR was observed, Fig. 6d.

PEGylation provided improved pharmacological properties *in vitro*. In media d_hyd_ and PDI of P_8K_NF suspensions were largely unchanged over the first 24 h, with a *c.*5% decrease in r_1_ but no measurable change in SAR. This suggests a window for retention of stealth properties that may be advantageous for retaining blood circulation following parenteral administration, see below. Over subsequent days d_hyd_ was found to increase to 72 nm with no change in PDI and no visible aggregation. There was a further 10% drop in relaxivity, compared to the aqueous suspension, but interestingly the SAR remained unchanged. We suggest that the increases in d_hyd_ are due to surface binding of biological molecules. However, this induced no significant changes in the SAR values; the heating is retained once particle aggregation is avoided for PNFs for which the Néel process determines the magnetic response.

The FFC-NMR profiles recorded before and after exposure to media for 39 days, Fig. S6, are almost unchanged, with a slight suppression at higher frequency. The low frequency (Néel part) is unchanged, so there is no change in τ _N_. This confirms the absence of aggregation and that the increase in d_hyd_ is due to bound biomolecules. The profile also confirms that the slight reduction in r_1_ measured at 40 MHz (Table 1) is real. Hence the Brownian r_1_ contribution is slightly reduced by surface bound bio-molecules, which we suggest is a screening effect. We have reported similar effects on r_1_ in FFC-NMR profiles of MNP clusters on adsorption of Au NPs onto the cluster surface [48].

Critically, the analysis demonstrates long-term stability of the suspensions and retention of magnetic properties over extended periods of time in complex media. PNF suspensions were also assessed in an adult, noncancerous, immortalised human kidney cell line HK-2 to gauge potential systemic toxicity, Fig. S7. No apparent cytotoxic effects were observed at Fe concentration up to 100 μg mL^−1^ (1.8 mM), a moderate dose-dependent decrease in cell viability was found at the highest concentrations (250–500 μg mL^−1^).

### 3.7. In vitro/vivo *MRI analysis*

Given that contrast enhancement achievable with any agent is a function of both it’s *in vivo* relaxivity and intra-tumour deposition, we tested the capability of P_8K_NF for generating contrast under T_2_-weighted conditions and its deposition in a pancreatic cancer model. We hypothesised that the low d_hyd_ and PEGylation would promote PNF accumulation in the tumour, and the stable oxide-gallol link would retain *in vivo* relaxivity. Therefore we evaluated the kinetics of PNF deposition and relaxivity in tumour. Feraheme^®^, or ferumoxytol (FMX), a clinically approved iron oxide nanoparticle treatment for anaemia in adults with chronic kidney disease [49] that has gained much attention for use in MRI as a T_2_ contrast agent [50,51], was used for comparison. On a 4.7 T (300 MHz) MR scanner high comparable r_2(*in vitro*)_ values (meaning in this case in suspension) of 52.4 and 66.1 mM^−1^ s^−1^ were recorded at 37°C for FMX and P_8K_NF suspensions, respectively, Fig. S8. MR images were recorded for consecutive slices of 1 mm thickness. Representative images in Fig, 7, upper panel grey scale, show signal intensity at a low echo time (TE value), giving maximum contrast. Visualisation was aided by generating maps of R_2_ values, calculated from the signal intensity in each voxel. Tumour R_2_ values were derived as an average of voxel-wise R_2_.

Intra tumour deposition of PNF was comparable to that of the clinically available nanoparticulate carrier. We employed fluorescently-labelled sterically stabilised liposomes (F-SSL, 100 nm, 1 μg lipid per animal, see Experimental) similar to Doxil^®^, as quantifiable probes of tissue-level deposition, and compared contrast-enhancement by PNF (∆R_2_) with tissue-level deposition of F-SSL (fluorescence microscopy) 24 h after co-administration of both agents. Co-administration enables direct comparison of deposition on an animal-by-animal basis. The studies were conducted in two PDX models differing significantly in vascular density and permeability [52], and in individual tumours across both models a positive linear correlation between F-SSL deposition and ∆R_2_ was observed, Fig, 7c, which suggests that PNF contrast-enhancement *in vivo* can predict macromolecular agent delivery in tumours. DCE-MRI perfusion scans with Gd-DTPA, a low molecular weight clinical contrast agent, were also performed on the same subjects as an added measure of the perfusion status of tumours. The Gd-DTPA enhancement showed no correlation with F-SSL deposition, Fig. S9, hence perfusion scans employing Gd-DTPA do not reflect tissue-level delivery of nanoparticulate agents.

Kinetic studies were conducted in mice implanted with the 18269 PDX model of pancreatic cancer. Timepoints were selected at 24 and 72 h post injection using the known biodistribution and pharmacokinetic modelling of FMX in man, reported by Fitzgerald et al. [53], where peak deposition in tumour tissue and blood plasma was achieved within several h of injection, with *c.*45% and *c.*90% clearance reported at 24 and 72 h post injection, respectively. In the baseline scan prior to PNF administration, the tumour appears blue, and R_2_ increased progressively from 24 to 72 h. Average ∆R_2_ values remained similar for 24 and 72 h (8.3 ± 4.3 and 8.5 ± 4.4 s^−1^, respectively), indicating stable intra-tumour r_2_ of P_8K_NF.

**Fig. 7.**
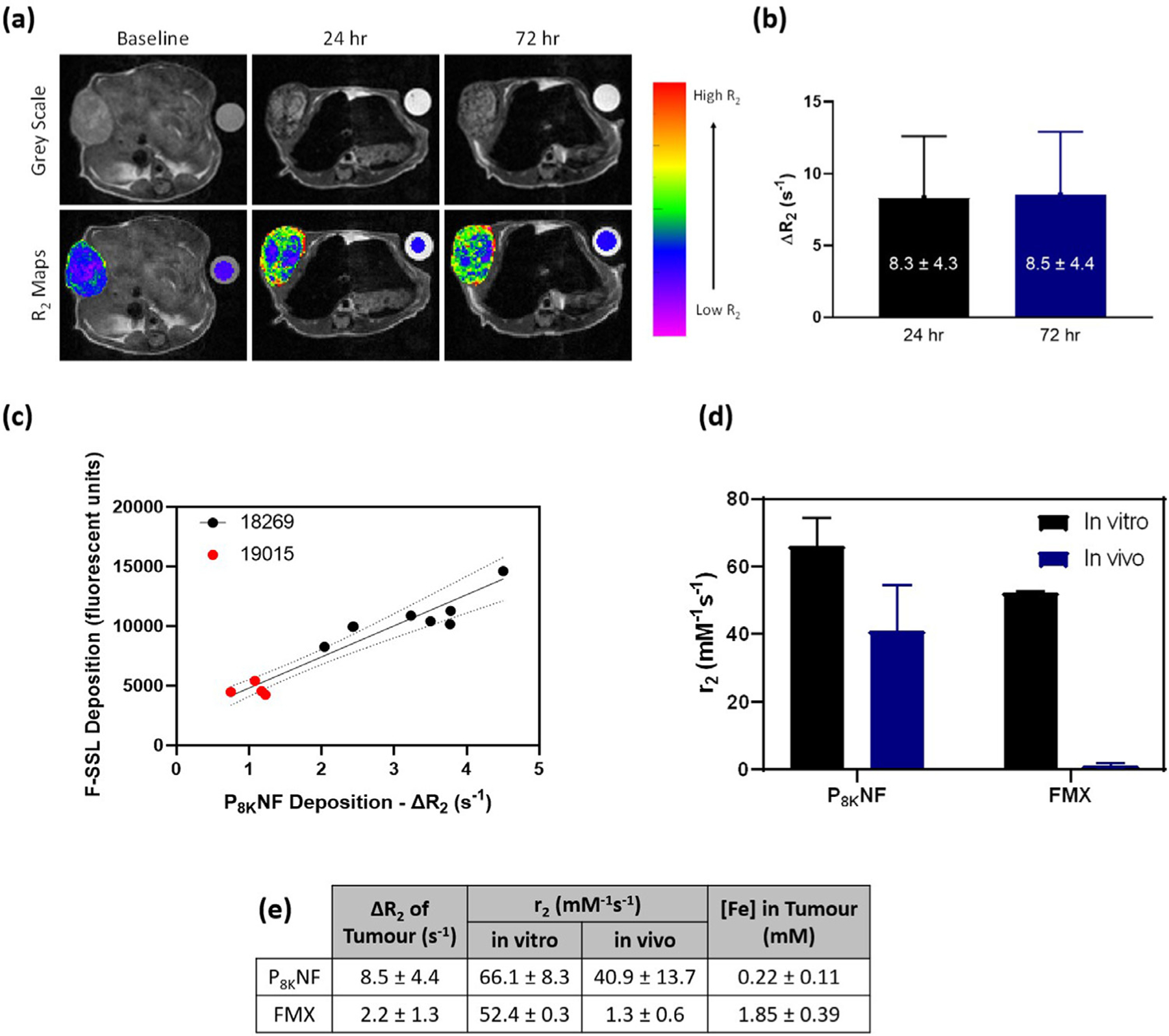
(a) Representative T_2_ -weighted MRI slices, with addition of R_2_ maps to aid in the visualisation of the observed change in contrast, for PNF treated group in the 18269 model at baseline, 24 and 72 h post injection (left to right, respectively) at a fixed TE value. (b) Average recorded ∆R_2_ for tumours in the P_8K_ NF treated group in the 18269 model at 24 and 72 h post injection. (c) Linear correlation of F-SSL deposition, quantified at the tissue-level using fluorescence microscopy, with recorded P_8K_ NF-enhanced ∆R_2_ 24 hour post injection in the 18269 and 19015 PDX models. (d) Comparison of r_2(*in vitro*)_ and r_2(*in vivo* at 72 h_) for P_8K_ NF and FMX. (e) Recorded ∆R_2_, r_2_ values and Fe concentration in tumour determined by ICP-AES, the latter was used to estimate r_2(*in vivo*)_.

In contrast, FMX deposition and r_2_ at 72 h (derived from ∆R_2_ and tumour Fe content, by ICP-AES) declined significantly, as suggested by the results summarised in Fig. 7e. A *c.*4-fold larger spatially-averaged ∆R_2_ value was determined for the P_8K_NF-, as compared to the FMX-treated, group despite the *c.*8-fold lower Fe content present. The data indicates that by 72 h the contrast generation of FMX is lost (*c.*44-fold decrease in r_2(*in vivo*)_, as compared to a *c.*1.7-fold decrease for P_8K_NF). Taken together the data in Fig. 7 show that the nanoflowers are retained intact in the tumour over a period of several days. We suggest that the gallol linker chemically stabilises the nanoflower cores which then retain their MRI response in the tumour micro-environment, mirroring the stability of nanoflowers in complex media *in vitro*. This also suggests that high hyperthermic efficiency may be maintained for extended time in the tumour microenvironment, which could provide possibilities for gated, repeatable hyperthermically-induced enhancement of local permeability, if not for localised AC-field ablation at this level of deposition. The fact that NFs can be stabilised with longer chain PEG opens a route to improving deposition. The preliminary *in vivo* study described here demonstrates potential for PNF suspensions as T_2_-contrast agents with good bio-stability and persistent magnetic responses following uptake, that can mark liposome deposition and may provide local hyperthermic hot-spots.

## 4. Conclusions

The biomaterials design presented, for preparation of PEGylated magnetic nanoflowers, provides highly stable suspensions that are fully dispersed for many weeks in complex media at high concentration. These advantages enabled exploitation of the favourable Néel-dominated magnetic properties of PNFs, opening up their potential both as potent mediators for AC-field hyperthermia and as contrast agents for T_2_-weighted MR imaging. The approach also enabled the first detailed evaluation of the effects of the immediate (ligands) and surrounding environment (isotonic suspension, or biological milieu) on these responses.

It is demonstrated that for PNFs, contrary to reports for related materials, both the SAR and the relaxivities are independent of the ligand shell under solvophilic conditions. The outstanding retention of SAR for multicore nanoflowers, even for long PEG grafts and in complex environments, arises because of the dominant Néel contribution which is insensitive to the surroundings. The relaxivity is unchanged because the strong steric stabilisation minimises inter-flower interactions, even in strong magnetic fields. It emerges that other interactions, often invoked to explain apparent ligand dependencies of r_1_, are of minor importance for NFs.

PNFs are shown to have T_2_-contrast efficacy *in vitro* comparable to the current generation of materials, but with good stability of the cores. The preliminary *in vivo* study strongly suggests unusual retention of contrast generation in the tumour microenvironment. The study demonstrates the potential of PEGylated nanoflower suspensions as T_2_-weighted MR imaging contrast agents for marking liposome deposition and for tumour imaging, which also have theragnostic potential as stable local hotspots for repeated AC-field stimulation.

## Supplementary Material

1

## Figures and Tables

**Fig. 1.**
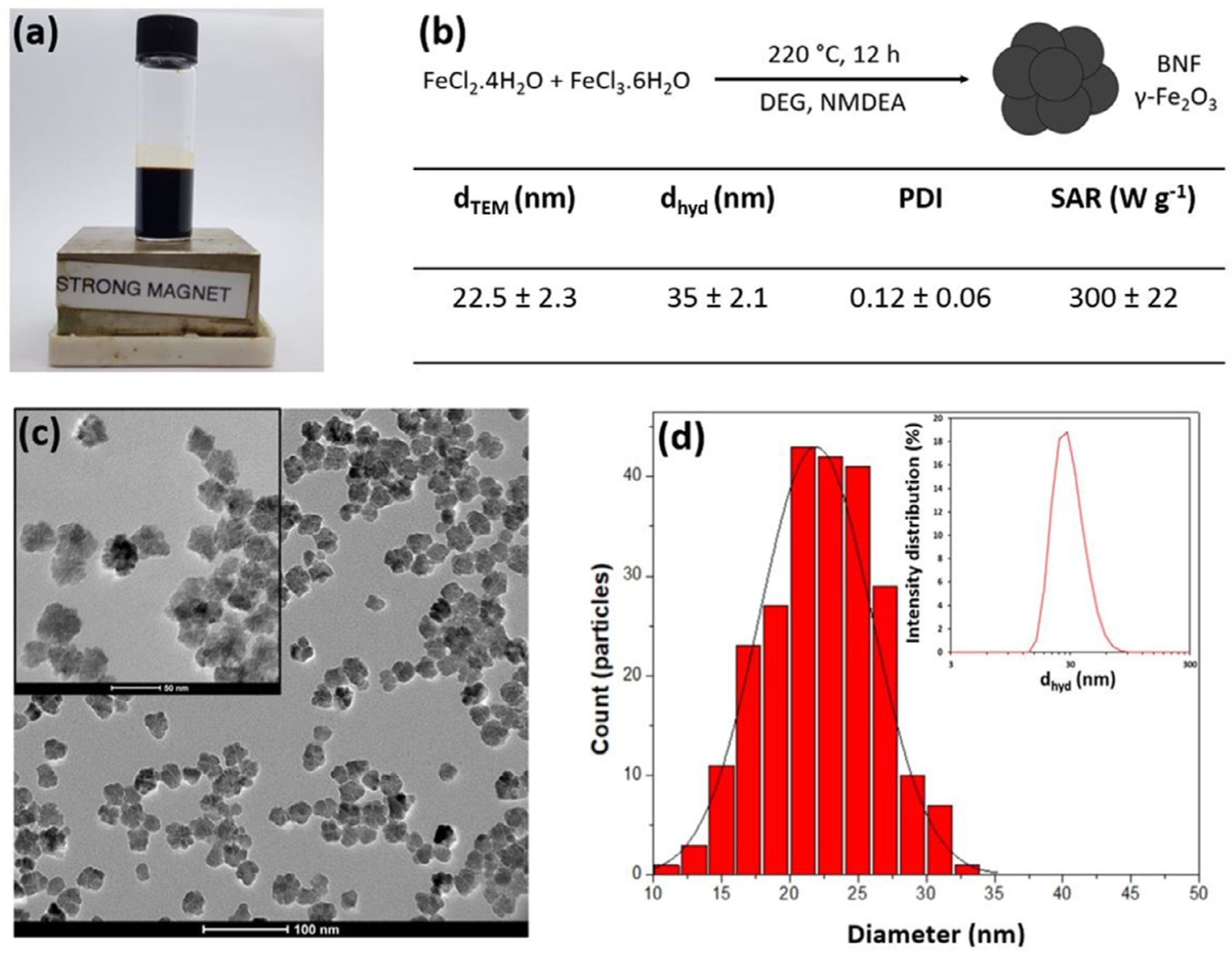
Uncoated nanoflower (BNF) characterisation. (a) A BNF suspension (250 mM, Fe concentration) placed on a permanent magnetic. (b) Summary of the physical properties of the BNFs and their aqueous suspensions, means and standard deviations for 10 standard syntheses are provided. (c) Representative TEM images of BNFs, scale bar 100 nm, inset; scale bar 50 nm. (d) Particle size distribution analysis (n = 250) of multiple TEM images (a log-normal fit is included for illustration) with, inset, a typical DLS intensity distribution recorded at pH 4 for which d_hyd_ 35 nm, PDI 0.12.

**Fig. 5.**
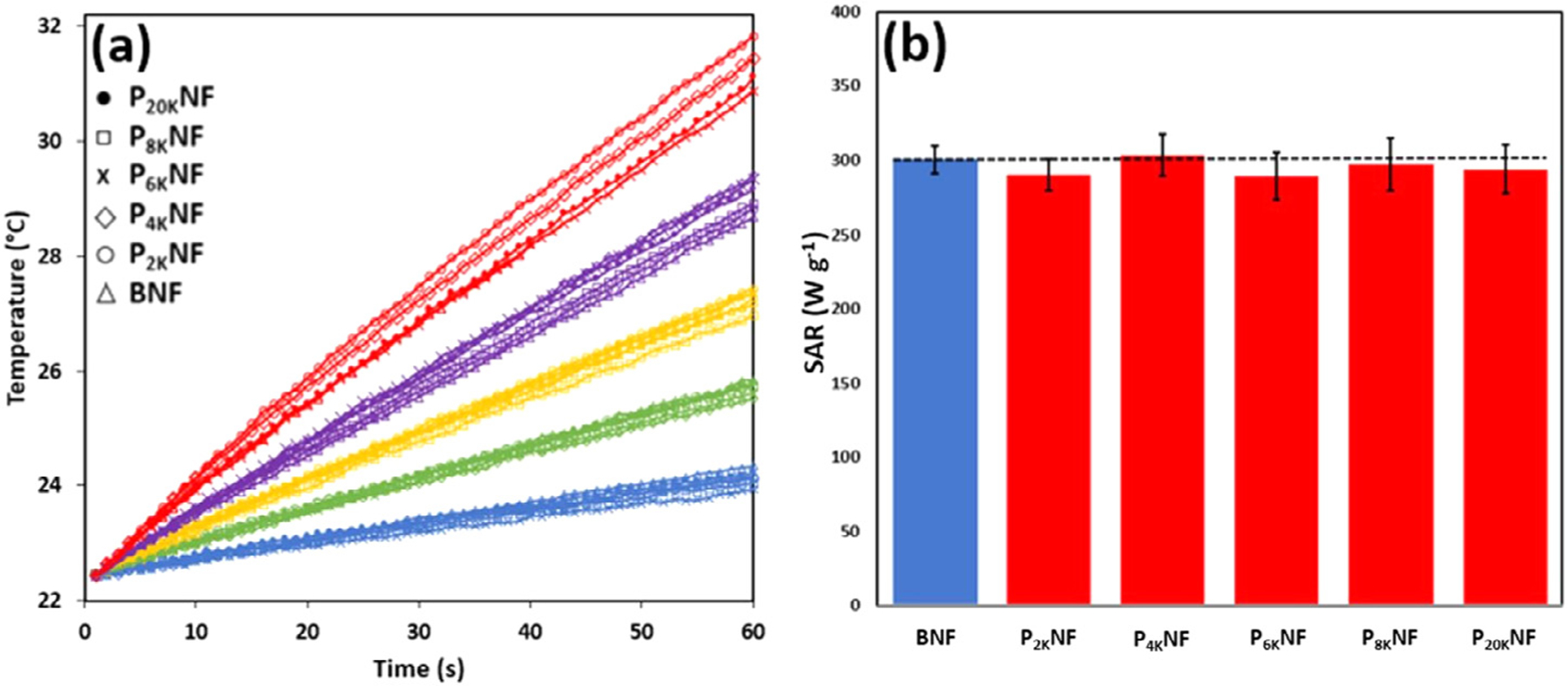
(a) AC-field induced heating response of BNF (pH 4) and PNF suspensions (pH 7) of varying chain length under an applied field strength of 15.9 kA m^−1^ and an AC frequency of 530 kHz, at Fe concentration 10 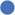, 20 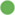, 30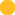, 40 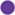, 50 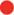 mM. Variability in SAR at each chain length is not concentration dependent, Fig. S5. (b) Corresponding SAR values, the error bars represent the average over all concentrations.

**Table 1:** Summary of the colloidal and magnetic properties of P_8K_ NF, Fe 1 mM (DLS, r_1_) and 50 mM Fe concentration (SAR), in both PBS and media over 39 days.

		t = 0	24 h	39 d
d_hyd_ (nm), [PDI]	**PBS**	56.4 [0.12]	54.4 [0.16]	56.8 [0.14]
	**Media**	56.2 [0.19]	57.0 [0.20]	71.2 [0.20]
r_1_ (s^−1^mM^−1^, 40 MHz)	**PBS**	16.4	16.1 (98%)	14.9 (91%)
	**Media**	16.3	15.5 (95%)	13.9 (85%)
SAR (W g^−1^)	**PBS**	291	290 (100%)	294 (101%)
	**Media**	288	282 (98%)	300 (104%)

## Statement of significance

Application of multicore magnetic iron-oxide nanoflowers (NFs) as efficient mediators for AC-field hyperthermia and as contrast agents for MR imaging has been limited by lack of colloidal stability in complex media and biosystems.

The optimized materials design presented is shown to reproducibly provide PEG grafted NF suspensions of exceptional colloidal stability in buffers and complex media, with significant hyperthermic and MRI utility which is unaffected by PEG length, anchoring group or bio-molecular adsorption.

Deposition in the selected pancreatic tumour model mirrors liposomal formulations providing a quantifiable probe of tissue-level liposome deposition and relaxivity is retained in the tumour microenvironment.

Hence the biomaterials design addresses the longstanding challenges of maintaining the *in vivo* magnetic efficiency of nanoparticles as theragnostic agents.
