# Supplementary material for: Formation of hydrated PEG layers on magnetic iron oxide nanoflowers shows internal magnetisation dynamics and generates high in-vivo efficacy for MRI and magnetic hyperthermia
Source: Acta Biomater. Author manuscript; Available in PMC 2023 Apr 28. (PMC10141539; doi:10.1016/j.actbio.2022.08.033)
Supplement: 1 [file NIHMS1886057-supplement-1.docx]

Supporting Information

**Formation of hydrated PEG layers on magnetic iron oxide nanoflowers shows internal magnetisation dynamics and generates high in-vivo efficacy for MRI and magnetic hyperthermia**

Eoin P. M^c^Kiernan, ^a^ Cara Moloney, ^a^ Tista Roy Chaudhuri, ^b^ Shane Clerkin, ^c^ Kevin Behan, ^a^ Robert M. Straubinger, ^b,d^ John Crean, ^c^ Dermot F. Brougham^a^*

^a^ School of Chemistry, University College Dublin, Belfield, Dublin 4, Ireland. *dermot.brougham@ucd.ie

^b^ Dept. of Pharmaceutical Sciences, School of Pharmacy and Pharmaceutical Sciences, University at Buffalo, State University of New York, Buffalo, NY, 14214, USA.

^c^ School of Biomolecular and Biomedical Science, Conway Institute, University College Dublin4, Ireland.

^d^ Department of Cell Stress Biology Roswell Park Comprehensive Cancer Center, Buffalo, NY 14263, USA.


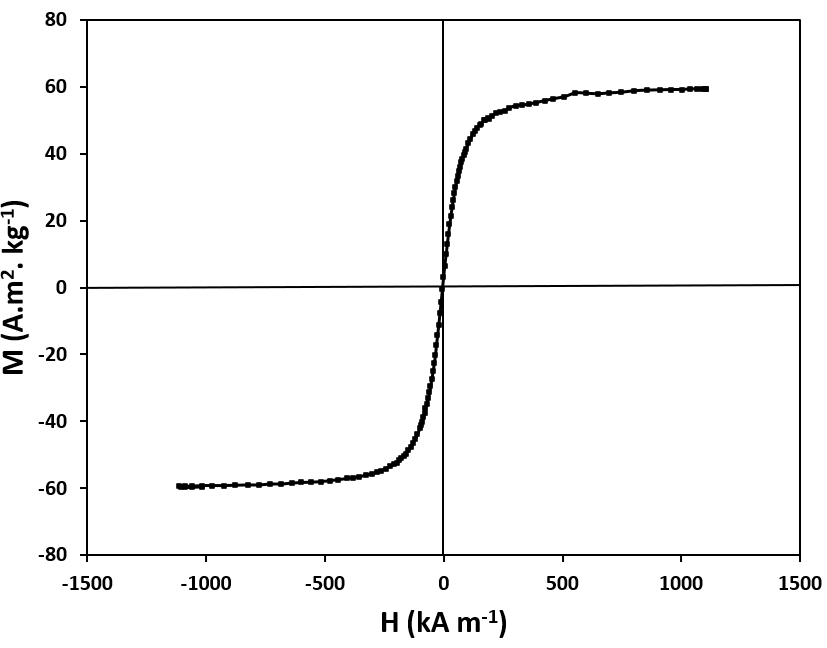


**Figure S1**. Field-dependent magnetization cycle at 298 K for BNFs showing no hysteresis.


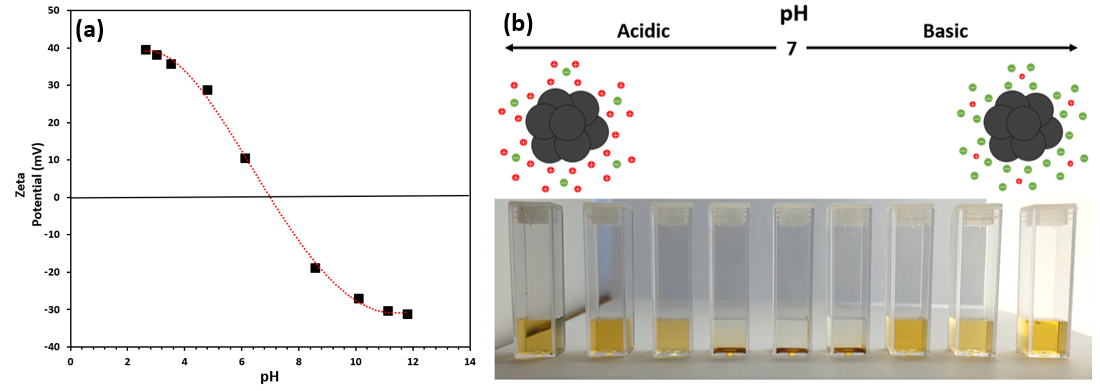


**Figure S2**. (a) Plot of zeta potential of BNFs vs the pH of the dispersion medium (water). (b) Image of BNF dispersions at varying pH after 24 h.

The isoelectric point of the nanoflowers, the pH at which the particle surface carries no net electrical charge is 7.2. This indicates the need for steric stabilization of the bare nanoflowers for in-vivo applications.


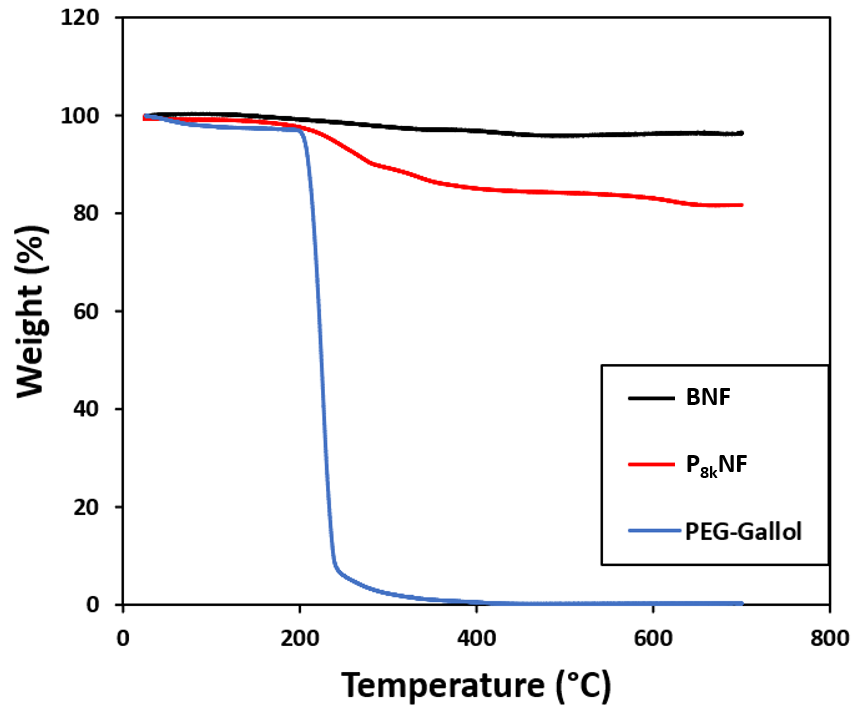


**Figure S3.** TGA responses recorded for dried BNF, P_8k_NF and PEG_8k_-Gallol sample, Weight (%) as a function of temperature (°C).

Weight loss of *c.*4% is observed for BNFs up to 700 °C, resulting from the loss of residual organic solvents from the preparation, which agrees with previously reported results by Bazzi et al.^17^ The recorded TGA curve for PEG-Gallol shows complete weight loss by 400 °C, which is expected for an organic compound which should completely degrade at elevated temperatures. For PNF, a further weight loss of *c.*14% is observed by 600 °C which is attributed to the degradation of bound PEG-Gallol to the NFs and the remaining 82% weight is attributed to the FeO content of the NFs which does not degrade at these temperatures.


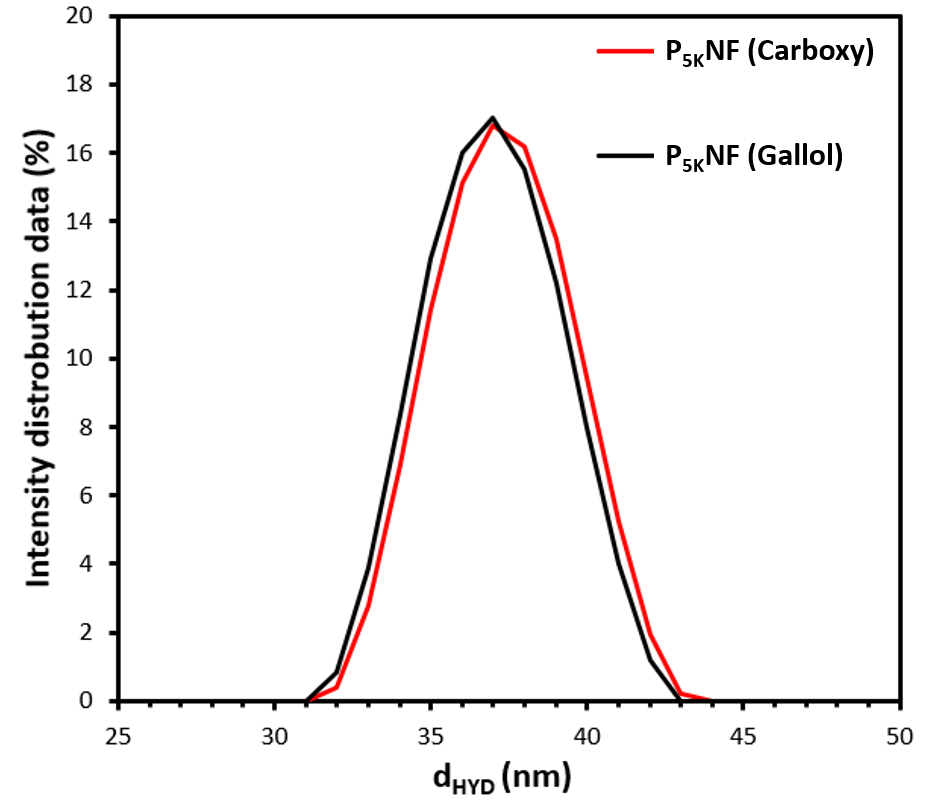


**Figure S4.** Size distribution by intensity for P_5K_NF suspensions with carboxy (d_hyd_ 58 nm, PDI 0.09) and gallol (d_hyd_ 54 nm, PDI 0.10) anchor groups.


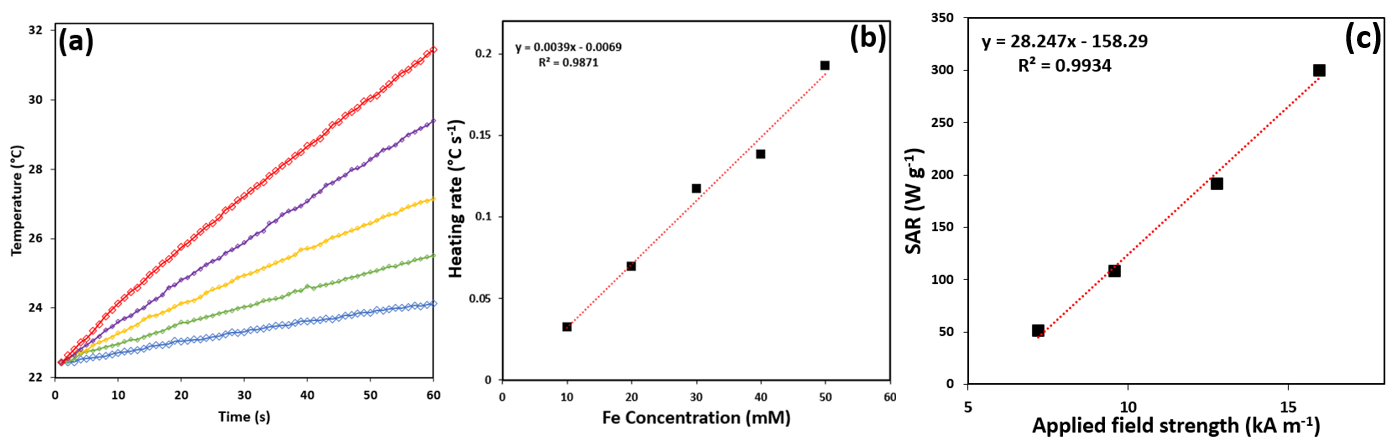
 **Figure S5. (a)** AC-field heating response of P_6k_NF suspensions under an applied field strength of 15.95 kA m^-1^ and an AC frequency of 530 kHz, at Fe concentrations of 10 ●, 20 ●, 30●, 40 ●, 50 ● mM. **(b)** Heating rate (°C s^-1^) of varying concentrations of the same P_6k_NF suspension. **(c)** SAR (W g^-1^) of the same P_6K_NF suspension as a function of magnetic field strength at 530 kHz.


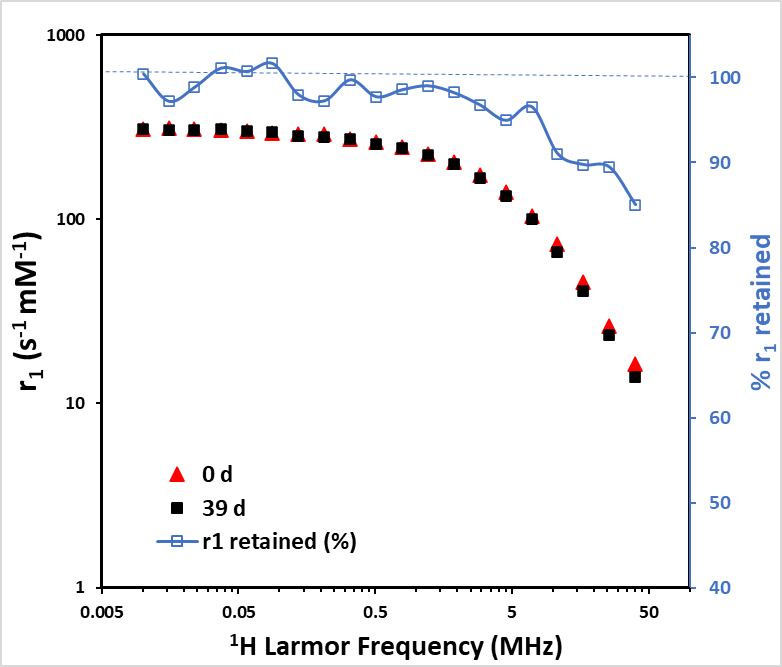


**Figure S6**. FFC-NMR profiles, recorded at 25 ^o^C, for P_8k_NF suspended in media for 0 and 39 days.

As shown in Figure S6, the profile is unchanged following long-term exposure to media for frequencies below 1-2 MHz, while above this range there is a relatively small gradual ongoing decrease in r_1_ with increasing frequency.

**

**Figure S7.** In vitro cytotoxicity of P_8K_NF in HK-2 cells. HK-2 cells were incubated for 48 h with various concentrations (25–500 µg mL^-1^) of P_8K_NF and viability was subsequently measured using the MTT colorimetric assay (n=4).

Cytotoxicity analysis was performed on P_8K_NF in order to evaluate its suitability for in-vivo analysis P_8K_NF demonstrated no apparent cytotoxic effects at a concentration of 100 µg mL^-1^ and below. There was a subsequent dose-dependent decrease in cell viability at higher concentrations (250–500 µg mL^-1^).


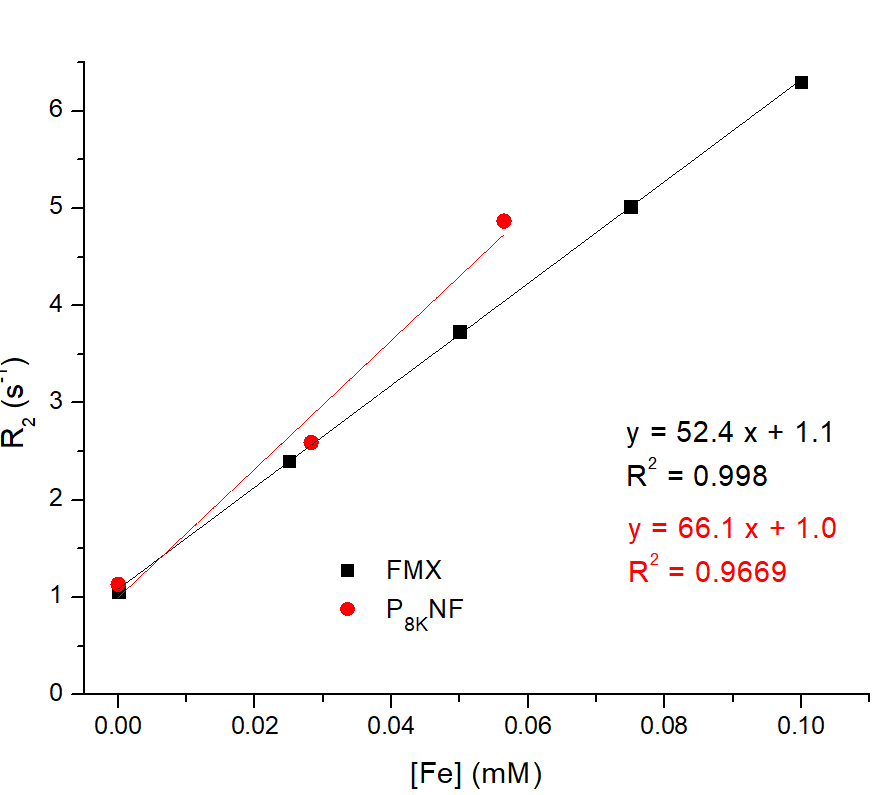


**Figure S8.** Concentration dependence of R_2_ for FMX and P_8K_NF recorded at 300 MHz, in the MR scanner, showing higher in vitro relaxivity (slope) for PNF suspensions.

Under the conditions used there was some colloidal instability in the very high (300 MHz) MRI field only for P_8K_NF at higher concentration. Hence the higher concentration points were not included in Figure s8. In the range below 0.06 mM the response was stable in the field and three data points are sufficient to confirm similar (if slightly higher) relaxivity for P_8K_NF. Note that colloidal instability in the field does not affect the outcomes/interpretation of the in vivo study as the injection was outside the field and immediate dilution (into the linear range) on injection would prevent any aggregation.


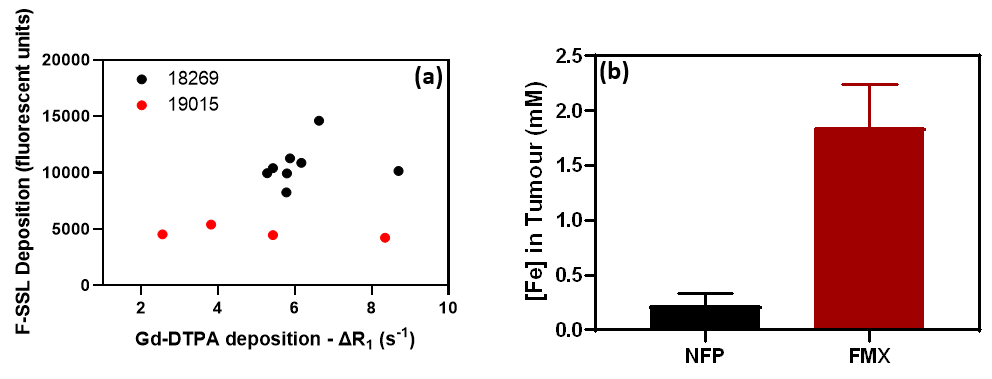


**Figure S9.** **(a)** Comparison of F-SSL deposition, quantified at the tissue-level using fluorescence microscopy, with recorded Gd-DTPA-enhanced ∆R_1_ values 24 hour post injection in the 18269 and 19015 PDX models, showing no correlation. **(b)** Comparison of P_8K_NF and FMX tumour deposition in 18269 PDX model 72 hours post injection.
